# 2,2′-[(1*E*,2*E*)-1,2-Bis(hy­droxy­imino)­ethane-1,2-di­yl]dipyridinium hexa­chloridorhenate(IV)

**DOI:** 10.1107/S1600536812035052

**Published:** 2012-08-15

**Authors:** Monika K. Krawczyk, Marta S. Krawczyk, Miłosz Siczek, Tadeusz Lis

**Affiliations:** a University of Wrocław, Faculty of Chemistry, 14 Joliot-Curie St, 50-383 Wrocław, Poland

## Abstract

The title salt, (C_12_H_12_N_4_O_2_)[ReCl_6_], consists of 2,2′-[(1*E*,2*E*)-1,2-bis­(hy­droxy­imino)­ethane-1,2-di­yl]dipyridinium cations and [ReCl_6_]^2−^ anions which are both located on inversion centres. Each cation consists of a glyoxime moiety attached to two protonated pyridine rings in *ortho* positions. In the crystal, *E*,*E* isomers of the cation are observed which differ in the spatial arrangement of the pyridine rings. These rings are positionally disordered over two positions with site-occupancy factors of 0.786 (7) and 0.214 (7). The geometry of the cation is compared with that of a recently reported dipyridyl­glyoxime with the same configuration. The cations and anions are involved in a network of inter­molecular O—H⋯Cl, N—H⋯Cl and C—H⋯Cl hydrogen bonds.

## Related literature

For the synthesis of K_2_[ReCl_6_], see: Enk (1931 ▶). For syntheses and research on dipyridyl­glyoxime, see: Soules *et al.* (1970 ▶); Richardson *et al.* (2002 ▶). For the structure of the [ReCl_6_]^2−^ ion, see: Takazawa *et al.* (1990 ▶); Hołyńska *et al.* (2007 ▶). For structures of rhenium compounds with monooximes, see: Jurisson *et al.* (1991 ▶, 1998 ▶). For background to π–π inter­actions, see: Janiak (2000 ▶). For related structures of the reported cation, see: Richardson & Steel (2000 ▶); Sabaté & Delalu (2012 ▶).
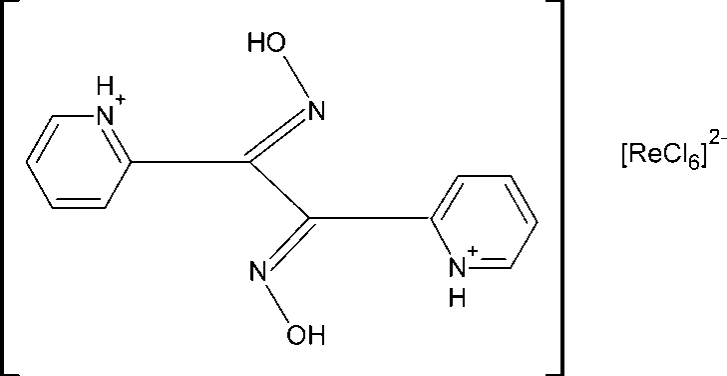


## Experimental

### 

#### Crystal data

(C_12_H_12_N_4_O_2_)[ReCl_6_]
*M* *_r_* = 643.16
Triclinic, 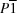

*a* = 7.836 (3) Å
*b* = 8.051 (3) Å
*c* = 8.733 (3) Å
α = 85.44 (3)°
β = 73.85 (3)°
γ = 65.12 (4)°
*V* = 479.6 (3) Å^3^
*Z* = 1
Mo *K*α radiation
μ = 7.18 mm^−1^
*T* = 100 K
0.15 × 0.06 × 0.02 mm

#### Data collection

Agilent Xcalibur PX KM-4-CCD diffractometer
Absorption correction: analytical (*CrysAlis PRO*; Agilent, 2011 ▶) *T*_min_ = 0.439, *T*_max_ = 0.841
9670 measured reflections
4738 independent reflections
4464 reflections with *I* > 2σ(*I*)
*R*_int_ = 0.026

#### Refinement

*R*[*F* ^2^ > 2σ(*F* ^2^)] = 0.025
*wR*(*F* ^2^) = 0.039
*S* = 1.02
4738 reflections
126 parameters
H-atom parameters constrained
Δρ_max_ = 1.41 e Å^−3^
Δρ_min_ = −1.02 e Å^−3^

### 

Data collection: *CrysAlis PRO* (Agilent, 2011 ▶); cell refinement: *CrysAlis PRO*; data reduction: *CrysAlis PRO*; program(s) used to solve structure: *SHELXS97* (Sheldrick, 2008 ▶); program(s) used to refine structure: *SHELXL97* (Sheldrick, 2008 ▶); molecular graphics: *XP* in *SHELXTL* (Sheldrick, 2008 ▶) and *DIAMOND* (Brandenburg, 2006 ▶); software used to prepare material for publication: *publCIF* (Westrip, 2010 ▶).

## Supplementary Material

Crystal structure: contains datablock(s) I, global. DOI: 10.1107/S1600536812035052/vn2047sup1.cif

Structure factors: contains datablock(s) I. DOI: 10.1107/S1600536812035052/vn2047Isup2.hkl

Additional supplementary materials:  crystallographic information; 3D view; checkCIF report

## Figures and Tables

**Table 1:** Hydrogen-bond geometry (Å, °)

*D*—H⋯*A*	*D*—H	H⋯*A*	*D*⋯*A*	*D*—H⋯*A*
O1—H1⋯Cl2^i^	0.84	2.43	3.2034 (19)	154
N12—H12⋯Cl2^ii^	0.88	2.44	3.318 (3)	179
N32—H32⋯Cl3^iii^	0.88	2.75	3.416 (9)	133
N32—H32⋯Cl1^iii^	0.88	2.81	3.495 (8)	136
C13—H13⋯Cl2^iv^	0.95	2.66	3.550 (4)	157
C16—H16⋯Cl3^iii^	0.95	2.79	3.658 (4)	152
C36—H36⋯Cl2^ii^	0.95	2.68	3.581 (7)	159

Symmetry codes: (i) 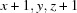; (ii) 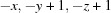; (iii) 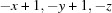; (iv) 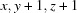.

**Table 2:** Selected inter­atomic distances (Å) and angles (°) for (C_12_H_12_N_4_O_2_)[ReCl_6_] and C_12_H_10_N_4_O_2_ (see also Sabaté & Delalu, 2012 ▶)

(C_12_H_12_N_4_O_2_)[ReCl_6_]		C_12_H_10_N_4_O_2_	
C1—C1^v^	1.472 (3)	C7—C7^ii^	1.468 (3)
C1—C11	1.482 (3)	C7—C2	1.501 (2)
C1—N1	1.290 (2)	C7—N8	1.289 (2)
N1—O1	1.3844 (19)	N8—O9	1.392 (2)
C1—N1—O1	111.88 (14)	C7—N8—O9	112.6 (1)
C1^v^—C1—N1	116.75 (18)	C7^ii^—C7—N8	116.0 (2)
C11—C1—C1^v^	119.1 (3)	C2—C7—C7^ii^	119.5 (1)
Re—Cl1	2.3500 (10)		
Re—Cl2	2.3707 (9)		
Re—Cl3	2.3531 (15)		

Symmetry codes: (ii) −*x*, 1 − *y*, 1 − *z*; (v) 1 − *x*, 1 − *y*, 1 − *z*.

**Fig. 1.**
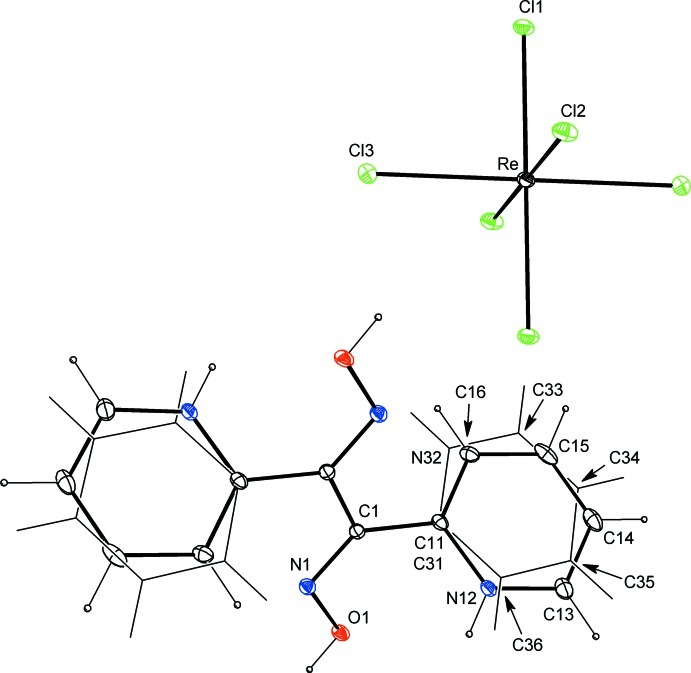
The crystal structure of (C12H12N4O2)[ReCl6] salt with labelling atoms in the asymmetric part of the unit cell. Displacement ellipsoids are drawn at the 20% probability level. The aromatic rings of minor-occupancy pyridyl component are drawn by thin lines.

**Fig. 2.**
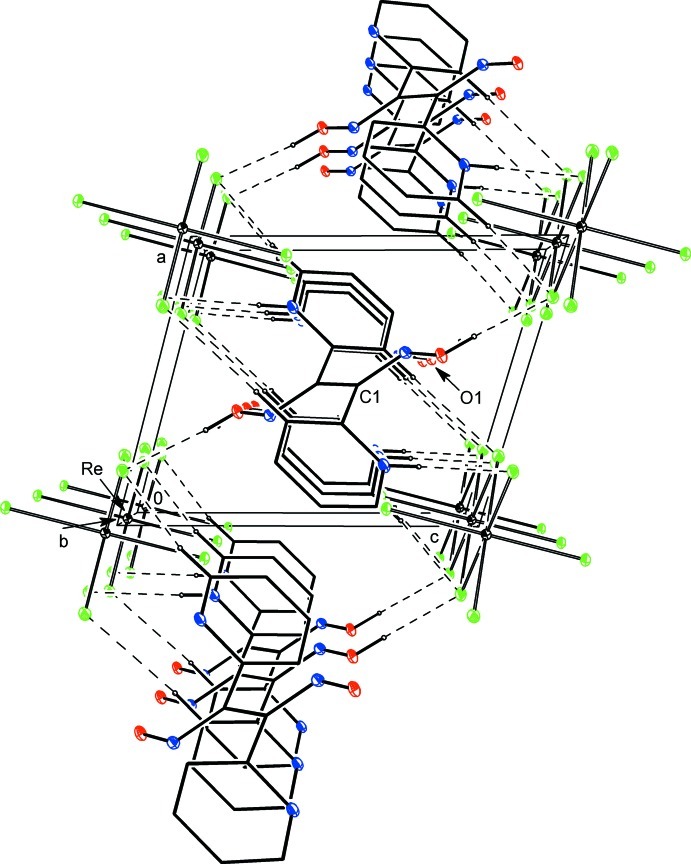
Packing diagram showing a layer architecture in the crystal structure of the title compound. Displacement ellipsoids are drawn at the 20% probability level. The aromatic rings of minor-occupancy component are omitted for clarity.

**Fig. 3.**
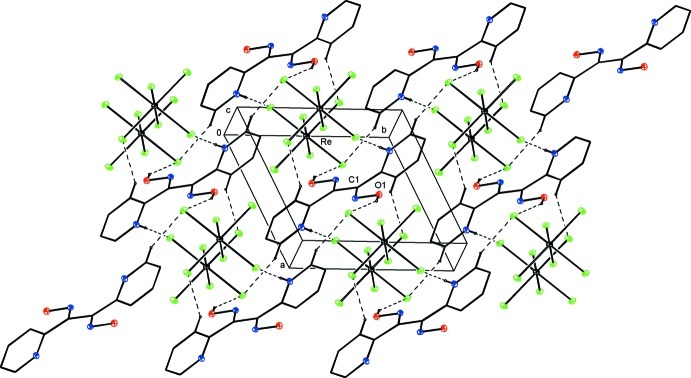
Ribbons of adjacent cations extending along [-111] direction which interact with [ReCl6]2- ions via O—H···Cl, N—H···Cl and C—H···Cl hydrogen bonds. Hydrogen atoms not involved in interactions are omitted for clarity. Displacement ellipsoids are drawn at the 20% probability level. The aromatic rings of minor-occupancy component are omitted for clarity.

**Fig. 4.**
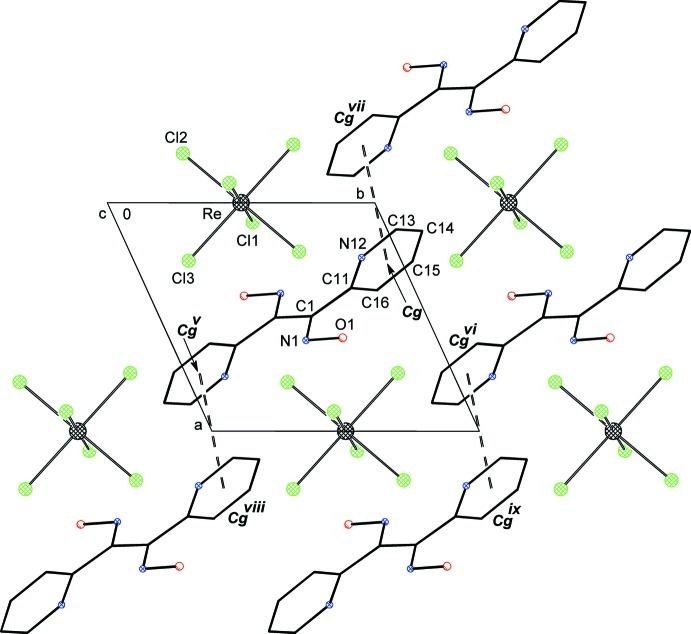
Packing diagram viewed down the c-axis showing π···π stacking interactions between the adjacent cations. The aromatic rings of minor-occupancy component are omitted for clarity. Symmetry codes: (v) 1–x, 1 - y, 1 - z; (vi) -x + 1, -y + 2, -z + 1; (vii) –x, -y + 2, -z + 1; (viii) x + 1, y - 1, z; (ix) x + 1, y, z.

## supplementary crystallographic information

### Comment

In the crystallographic literature many metal complexes with dioximes have been reported. In the case of rhenium compounds there are three crystal structures with coordinated monooximes (Jurisson *et al.*, 1991; Jurisson *et al.*, 1998), while the complexes of Re with dioximes were not reported. Therefore, it seemed an attractive idea to obtain new compounds of rhenium with dioximes. As a result of our initial attempts we obtained a new rhenium salt of the formula (C_12_H_12_N_4_O_2_)[ReCl_6_]. The cation is a protonated form of (1*E*,2*E)*-2,2'-dipyridylglyoxime, C_12_H_10_N_4_O_2_ [or (1*E*,2*E*)-*N*,*N*'-dihydroxy-1,2-di(pyridin-2-yl)ethane-1,2-diimine or (1*E*,2*E)*-1,2-di(pyridin-2-yl)ethanedione dioxime]. The crystal structure of (1*E*,2*E)*-2,2'-dipyridylglyoxime was recently reported by Sabaté & Delalu (2012). In the literature there is known one structure which contains a cyclic form of the C_12_H_10_N_4_O_2_ dioxime as a di(2-pyridyl)furoxan coordinated to copper(II) (Richardson & Steel, 2000). The C_12_H_10_N_4_O_2_ dioxime was initially obtained by Soules *et al.*, (1970). Another preparating method was published by Richardson *et al.*, (2002).

In the crystal structure reported here both cation and anion are centrosymmetric. The cation is built up by the planar glyoxime fragment, HO—N=C—C=N—OH and two protonated pyridyl rings. The pyridyl groups are attached to the dioxime unit *via* C atoms located at the *ortho* positions with respect to the N atoms. The (C_12_H_12_N_4_O_2_)^2+^ unit may adopt many geometries because of the conformations of the glyoxime moiety (resulting in *E,E, E,Z* or *Z,Z* isomers) and spatial arrangement of pyridyl rings in relation to the HO—N=C—C=N—OH fragment. In the studied crystal structure the cations are the *E,E* isomers where the glyoxime moieties form interplanar angles of about 55° with planes of aromatic rings*.* Similar values of the geometrical parameters are observed compared to those published by Sabaté & Delalu (2012) (Table 2) for (1*E*,2*E)*-2,2'-dipyridylglyoxime. However slight differences in overall geometries can be distinguished. Contrary to the studied cation, in the dioxime structure published previously the planar HO—N=C—C=N—OH unit is perpendicular to the planes of aromatic rings. The results reported here for the rhenium salt reveal that the presence of H atoms bonded to N atoms in aromatic rings involved in N—H···Cl hydrogen bonds (Table 1) as well as the mutual arrangement of (C_12_H_12_N_4_O_2_)^2+^ and [ReCl_6_]^2-^ ions influence the overall geometry of the cation compared to dipyridylglyoxime.

As was mentioned before, the two pyridyl rings are bonded *via* C atoms in *ortho* positions to the planar glyoxime moiety (*viz.* to C1 atoms). The aromatic rings are disordered over two positions and turned to each other by an angle of about 180° (Fig. 1). The pyridyl rings of the major-occupancy form an interplanar angle of 55.4 (1)° with the glyoxime unit, while the aromatic rings of the minor-occupancy form an angle of 60.2 (3)°. The observed disorder can be interpreted as a solid solution of isomers of the cation due to the arrangement of pyridyl groups, where the glyoxime units have the same geometry. The disorder can be considered in three ways. In the first case the two centrosymmetric conformers can be in the crystal which occur in 78 and 22%. In the second one the three isomers can be observed: one centrosymmetric in 56% and two noncentrosymmetric both in 22%. In the third case the four conformers can be considered: two centrosymmetric and two noncentrosymmetric. In all the cases the preferred conformer is the centrosymmetric one for which the pyridyl rings form the interplanar angle of 55.4 (1)° with HO—N=C—C=N—OH entity.

In the title compound the hexachloridorhenate(IV) counterion has a slightly distorted octahedral geometry. Two of the three crystallographically independent Re—Cl bond lengths [2.3500 (10) and 2.3531 (15) Å] are comparable with 2.3545 (9) Å in [ReCl_6_]^2-^ published by Takazawa *et al.*, (1990). The third one is longer [2.3707 (9) Å] which can be explained by the involvement of Cl atom in the O—H···Cl and N—H···Cl hydrogen bonds (Table 1). This value is slightly longer than the Re—Cl bond lenghts [2.3604 (11) and 2.3644 (12) Å] for [ReCl_6_]^2-^ published by Hołyńska *et al.*, (2007) where only weak C—H···Cl hydrogen bonds are observed.

The crystal structure of (C_12_H_12_N_4_O_2_)[ReCl_6_] is stabilized by a network of O—H···Cl, N—H···Cl and C—H···Cl hydrogen bonds (Table 1) as well as π···π stacking interactions of parallel displaced pyridyl rings (Janiak 2000). The studied crystal structure reveals a layer architecture viewed down the [010] direction (Fig. 2). The cations are arranged in ribbons extended along the [-111] direction and interact with [ReCl_6_]^2-^ ions *via* hydrogen bonds (Table 1, Fig. 3). The (C_12_H_12_N_4_O_2_)^2+^ ions within ribbons are also involved in π···π interactions with centroid-centroid distance of 3.878 (2) Å and an slip angle of 26.99 (1)° (*Cg*···*Cg*^vii^, *Cg* = C11/N12/C13/C14/C15/C16, (vii)–x, -*y* + 2, -*z* + 1) (calculated with *DIAMOND* (Brandenburg, 2006)) (Fig. 4).

### Experimental

(1*E*,2*E*)-2,2'-dipyridylglyoxime, C_12_H_10_N_4_O_2_ used for the synthesis was obtained according to the method reported by Richardson *et al.*, (2002). The K_2_[ReCl_6_] was obtained as published previously (Enk, 1931). The potassium hexachloridorhenate was mixed with C_12_H_10_N_4_O_2_ and acetic acid (molar ratio 1:1:3, respectively) and refluxed in water for 3 h at about 100 °C. The mixture was allowed to evaporate in air to give brown plate crystals.

### Refinement

The H atoms of the N—H and O—H groups were located in difference Fourier maps but were introduced in positions calculated from geometry, with N—H = 0.88 Å and *U*_iso_(H) = 1.2*U*_eq_(N) and O—H = 0.84 Å and *U*_iso_(H) = 1.5*U*_eq_(O). The disorder of the pyridyl rings was modelled over two positions, with occupancies of 0.786 (7) and 0.214 (7); the minor-occupancy component was refined isotropically. The disorder of the aromatic ring of the minor-occupancy components was modelled using AFIX 66. EADP constrains were used for three pairs of disordered atoms: C11 and C31; N12 and C36, C16 and N32. The H atoms of aromatic rings were treated as riding atoms in geometrically idealized positions, with C—H = 0.95 Å and *U*_iso_(H) = 1.2*U*_eq_(C). The highest residual electron density of 1.40 e Å^-3^ was located 0.80 Å from Re; the deepest hole of -1.02 e Å^-3^ was located 0.62 Å from Re.

### Crystal data

**Table:**

(C_12_H_12_N_4_O_2_)[ReCl_6_]	*Z* = 1
*M**_r_* = 643.16	*F*(000) = 305
Triclinic, *P*1	*D*_x_ = 2.227 Mg m^−^^3^
Hall symbol: -P 1	Mo *K*α radiation, λ = 0.71073 Å
*a* = 7.836 (3) Å	Cell parameters from 10732 reflections
*b* = 8.051 (3) Å	θ = 2.8–38.5°
*c* = 8.733 (3) Å	µ = 7.18 mm^−^^1^
α = 85.44 (3)°	*T* = 100 K
β = 73.85 (3)°	Plate, brown
γ = 65.12 (4)°	0.15 × 0.06 × 0.02 mm
*V* = 479.6 (3) Å^3^	

### Data collection

**Table:**

Agilent Xcalibur PX KM-4-CCD diffractometer	4738 independent reflections
Radiation source: fine-focus sealed tube	4464 reflections with *I* > 2σ(*I*)
Graphite monochromator	*R*_int_ = 0.026
φ and ω scans	θ_max_ = 38.5°, θ_min_ = 2.8°
Absorption correction: analytical (*CrysAlis PRO*; Agilent, 2011)	*h* = −13→13
*T*_min_ = 0.439, *T*_max_ = 0.841	*k* = −14→14
9670 measured reflections	*l* = −15→12

### Refinement

**Table:**

Refinement on *F*^2^	Primary atom site location: structure-invariant direct methods
Least-squares matrix: full	Secondary atom site location: difference Fourier map
*R*[*F*^2^ > 2σ(*F*^2^)] = 0.025	Hydrogen site location: inferred from neighbouring sites
*wR*(*F*^2^) = 0.039	H-atom parameters constrained
*S* = 1.02	*w* = 1/[σ^2^(*F*_o_^2^) + (0.012*P*)^2^] where *P* = (*F*_o_^2^ + 2*F*_c_^2^)/3
4738 reflections	(Δ/σ)_max_ < 0.001
126 parameters	Δρ_max_ = 1.41 e Å^−^^3^
0 restraints	Δρ_min_ = −1.02 e Å^−^^3^

### Special details

**Table:**

Geometry. All e.s.d.'s (except the e.s.d. in the dihedral angle between two l.s. planes) are estimated using the full covariance matrix. The cell e.s.d.'s are taken into account individually in the estimation of e.s.d.'s in distances, angles and torsion angles; correlations between e.s.d.'s in cell parameters are only used when they are defined by crystal symmetry. An approximate (isotropic) treatment of cell e.s.d.'s is used for estimating e.s.d.'s involving l.s. planes.
Refinement. Refinement of *F*^2^ against ALL reflections. The weighted *R*-factor *wR* and goodness of fit *S* are based on *F*^2^, conventional *R*-factors *R* are based on *F*, with *F* set to zero for negative *F*^2^. The threshold expression of *F*^2^ > σ(*F*^2^) is used only for calculating *R*-factors(gt) *etc*. and is not relevant to the choice of reflections for refinement. *R*-factors based on *F*^2^ are statistically about twice as large as those based on *F*, and *R*- factors based on ALL data will be even larger.

### Fractional atomic coordinates and isotropic or equivalent isotropic displacement parameters (Å^2^)

**Table:**

	*x*	*y*	*z*	*U*_iso_*/*U*_eq_	Occ. (<1)
Re	0.0000	0.5000	0.0000	0.01565 (3)	
Cl1	0.08601 (6)	0.50992 (6)	−0.27962 (5)	0.01953 (8)	
Cl2	−0.21489 (7)	0.36685 (6)	−0.01114 (5)	0.02267 (9)	
Cl3	0.25414 (7)	0.20532 (6)	−0.01585 (5)	0.02528 (10)	
O1	0.5915 (2)	0.64822 (17)	0.74131 (15)	0.0205 (3)	
H1	0.6525	0.6038	0.8106	0.031*	
N1	0.6027 (2)	0.5076 (2)	0.65197 (18)	0.0178 (3)	
C1	0.4972 (2)	0.5685 (2)	0.55275 (19)	0.0151 (3)	
C11	0.3742 (6)	0.7650 (4)	0.5391 (5)	0.0171 (3)	0.786 (7)
N12	0.2411 (4)	0.8555 (4)	0.6748 (3)	0.0190 (4)	0.786 (7)
H12	0.2360	0.7952	0.7633	0.023*	0.786 (7)
C13	0.1165 (4)	1.0326 (4)	0.6810 (4)	0.0283 (7)	0.786 (7)
H13	0.0257	1.0908	0.7789	0.034*	0.786 (7)
C14	0.1219 (5)	1.1290 (3)	0.5432 (4)	0.0297 (8)	0.786 (7)
H14	0.0345	1.2543	0.5449	0.036*	0.786 (7)
C15	0.2549 (5)	1.0417 (4)	0.4039 (4)	0.0285 (8)	0.786 (7)
H15	0.2595	1.1070	0.3084	0.034*	0.786 (7)
C16	0.3830 (6)	0.8590 (5)	0.4009 (4)	0.0217 (6)	0.786 (7)
H16	0.4761	0.7994	0.3042	0.026*	0.786 (7)
C31	0.377 (2)	0.7638 (9)	0.5356 (14)	0.0171 (3)	0.214 (7)
N32	0.4339 (15)	0.8257 (9)	0.3851 (11)	0.0217 (6)	0.214 (7)
H32	0.5283	0.7485	0.3097	0.026*	0.214 (7)
C33	0.3418 (12)	1.0095 (10)	0.3537 (8)	0.0182 (19)*	0.214 (7)
H33	0.3809	1.0518	0.2509	0.022*	0.214 (7)
C34	0.1926 (11)	1.1314 (7)	0.4728 (10)	0.0153 (18)*	0.214 (7)
H34	0.1297	1.2570	0.4514	0.018*	0.214 (7)
C35	0.1355 (11)	1.0695 (11)	0.6233 (9)	0.0157 (19)*	0.214 (7)
H35	0.0335	1.1528	0.7047	0.019*	0.214 (7)
C36	0.2275 (18)	0.8857 (13)	0.6547 (10)	0.0190 (4)	0.214 (7)
H36	0.1884	0.8434	0.7576	0.023*	0.214 (7)

### Atomic displacement parameters (Å^2^)

**Table:**

	*U*^11^	*U*^22^	*U*^33^	*U*^12^	*U*^13^	*U*^23^
Re	0.01360 (4)	0.02072 (5)	0.01234 (5)	−0.00645 (3)	−0.00438 (3)	0.00154 (3)
Cl1	0.02003 (19)	0.0271 (2)	0.01245 (18)	−0.01074 (16)	−0.00483 (15)	0.00221 (15)
Cl2	0.0230 (2)	0.0342 (2)	0.0169 (2)	−0.01699 (18)	−0.00705 (16)	0.00295 (16)
Cl3	0.0234 (2)	0.0247 (2)	0.0201 (2)	−0.00156 (17)	−0.00808 (17)	0.00118 (16)
O1	0.0239 (6)	0.0236 (6)	0.0188 (6)	−0.0106 (5)	−0.0120 (5)	0.0014 (5)
N1	0.0173 (7)	0.0194 (7)	0.0177 (7)	−0.0074 (5)	−0.0066 (5)	0.0018 (5)
C1	0.0135 (7)	0.0167 (7)	0.0148 (7)	−0.0067 (6)	−0.0033 (6)	0.0029 (6)
C11	0.0195 (8)	0.0175 (8)	0.0181 (8)	−0.0085 (6)	−0.0102 (6)	0.0026 (6)
N12	0.0214 (9)	0.0172 (11)	0.0181 (10)	−0.0045 (8)	−0.0108 (7)	0.0016 (7)
C13	0.0334 (14)	0.0195 (12)	0.0331 (17)	−0.0047 (10)	−0.0195 (12)	−0.0023 (11)
C14	0.0407 (18)	0.0164 (11)	0.0398 (19)	−0.0098 (10)	−0.0273 (16)	0.0049 (10)
C15	0.042 (2)	0.0273 (14)	0.0336 (17)	−0.0230 (14)	−0.0261 (16)	0.0176 (13)
C16	0.0285 (18)	0.0218 (13)	0.0174 (11)	−0.0124 (12)	−0.0083 (12)	0.0053 (9)
C31	0.0195 (8)	0.0175 (8)	0.0181 (8)	−0.0085 (6)	−0.0102 (6)	0.0026 (6)
N32	0.0285 (18)	0.0218 (13)	0.0174 (11)	−0.0124 (12)	−0.0083 (12)	0.0053 (9)
C36	0.0214 (9)	0.0172 (11)	0.0181 (10)	−0.0045 (8)	−0.0108 (7)	0.0016 (7)

### Geometric parameters (Å, º)

**Table:**

Re—Cl1	2.3500 (10)	C14—H14	0.9500
Re—Cl2	2.3707 (9)	C15—C16	1.386 (4)
Re—Cl3	2.3531 (15)	C15—H15	0.9500
O1—N1	1.3844 (19)	C16—H16	0.9500
O1—H1	0.8400	C31—N32	1.3900
N1—C1	1.290 (2)	C31—C36	1.3900
C1—C1^i^	1.472 (3)	N32—C33	1.3900
C1—C11	1.482 (3)	N32—H32	0.8800
C1—C31	1.476 (6)	C33—C34	1.3900
C11—N12	1.354 (3)	C33—H33	0.9500
C11—C16	1.374 (3)	C34—C35	1.3900
N12—C13	1.342 (3)	C34—H34	0.9500
N12—H12	0.8800	C35—C36	1.3900
C13—C14	1.379 (4)	C35—H35	0.9500
C13—H13	0.9500	C36—H36	0.9500
C14—C15	1.369 (4)		
			
Cl1—Re—Cl3	90.35 (4)	C13—N12—C11	123.2 (2)
Cl1—Re—Cl2	90.11 (3)	N12—C13—C14	119.1 (3)
Cl3—Re—Cl2	89.72 (4)	C15—C14—C13	119.1 (2)
N1—O1—H1	109.5	C14—C15—C16	120.6 (2)
C1—N1—O1	111.88 (14)	C11—C16—C15	119.4 (3)
N1—C1—C1^i^	116.75 (18)	N32—C31—C36	120.0
N1—C1—C11	124.1 (2)	N32—C31—C1	113.3 (6)
C1^i^—C1—C11	119.1 (3)	C36—C31—C1	126.6 (7)
N1—C1—C31	124.8 (7)	C31—N32—C33	120.0
C1^i^—C1—C31	118.4 (7)	C34—C33—N32	120.0
N12—C11—C16	118.5 (2)	C35—C34—C33	120.0
N12—C11—C1	116.2 (3)	C34—C35—C36	120.0
C16—C11—C1	125.3 (3)	C35—C36—C31	120.0
			
O1—N1—C1—C1^i^	178.00 (16)	C1—C11—C16—C15	178.0 (4)
O1—N1—C1—C11	−1.6 (3)	N1—C1—C31—N32	118.0 (6)
O1—N1—C1—C31	−0.5 (6)	C1^i^—C1—C31—N32	−60.4 (9)
N1—C1—C11—N12	−55.6 (4)	N1—C1—C31—C36	−57.8 (11)
C1^i^—C1—C11—N12	124.8 (3)	C1^i^—C1—C31—C36	123.7 (7)
N1—C1—C11—C16	125.6 (3)	C1—C31—N32—C33	−176.1 (13)
C1^i^—C1—C11—C16	−54.0 (5)	C1—C31—C36—C35	175.6 (15)
C1—C11—N12—C13	−178.6 (4)		

Symmetry code: (i) −*x*+1, −*y*+1, −*z*+1.

### Hydrogen-bond geometry (Å, º)

**Table:**

*D*—H···*A*	*D*—H	H···*A*	*D*···*A*	*D*—H···*A*
O1—H1···Cl2^ii^	0.84	2.43	3.2034 (19)	154
N12—H12···Cl2^iii^	0.88	2.44	3.318 (3)	179
N32—H32···Cl3^iv^	0.88	2.75	3.416 (9)	133
N32—H32···Cl1^iv^	0.88	2.81	3.495 (8)	136
C13—H13···Cl2^v^	0.95	2.66	3.550 (4)	157
C16—H16···Cl3^iv^	0.95	2.79	3.658 (4)	152
C36—H36···Cl2^iii^	0.95	2.68	3.581 (7)	159

Symmetry codes: (ii) *x*+1, *y*, *z*+1; (iii) −*x*, −*y*+1, −*z*+1; (iv) −*x*+1, −*y*+1, −*z*; (v) *x*, *y*+1, *z*+1.

### Selected interatomic distances (Å) and angles (°) for (C_12_H_12_N_4_O_2_)[ReCl_6_] and C_12_H_10_N_4_O_2_ (see also Sabaté &amp; Delalu, 2012).

**Table:**

(C_12_H_12_N_4_O_2_)[ReCl_6_]		C_12_H_10_N_4_O_2_	
C1—C1^v^	1.472 (3)	C7—C7^ii^	1.468 (3)
C1—C11	1.482 (3)	C7—C2	1.501 (2)
C1—N1	1.290 (2)	C7—N8	1.289 (2)
N1—O1	1.3844 (19)	N8—O9	1.392 (2)
C1—N1—O1	111.88 (14)	C7—N8—O9	112.6 (1)
C1^v^—C1—N1	116.75 (18)	C7^ii^—C7—N8	116.0 (2)
C11—C1—C1^v^	119.1 (3)	C2—C7—C7^ii^	119.5 (1)
Re—Cl1	2.3500 (10)		
Re—Cl2	2.3707 (9)		
Re—Cl3	2.3531 (15)		

Notes: Symmetry codes: (ii) -x, 1-y, 1-z; (v) 1-x, 1-y, 1-z.
